# Stability of clonidine hydrochloride in an oral powder form compounded for pediatric patients in Japan

**DOI:** 10.1186/s40780-021-00214-x

**Published:** 2021-09-01

**Authors:** Jumpei Saito, Takehisa Hanawa, Takahiro Matsumoto, Nozomi Yoshikawa, Tsutomu Harada, Kana Iwahashi, Hidefumi Nakamura, Akimasa Yamatani

**Affiliations:** 1 Department of Pharmacy, National Center for Child Health and Development, 2-10-1 Okura, Setagaya-ku, Tokyo, 157-8535 Japan; 2 Division of Clinical Pharmacology and Oral Formulation Development, National Center for Child Health and Development, 2-10-1 Okura, Setagaya-ku, Tokyo, 157-8535 Japan; 3 Faculty of Pharmaceutical Sciences, Tokyo University of Science, 2641 Yamazaki, Noda-shi, Chiba, 278-8510 Japan; 4 R&D center, Ohara Pharmaceutical Co.,Ltd., Shiga, Japan; 5 Division of Pharmaceutics, Showa University, 1-5-8 Hatanodai, Shinagawa-ku, Tokyo, 142-8555 Japan; 6 Department of Research and Development Supervision, National Center for Child Health and Development, 2-10-1 Okura, Setagaya-ku, Tokyo, 157-8535 Japan

**Keywords:** Compounding, Pediatric patients, Clonidine hydrochloride, Oral powder form, Stability

## Abstract

**Background:**

Clonidine hydrochloride is used to treat sedative agent withdrawals, malignant hypertension, and anesthesia complications. Clonidine is also prescribed off-label to pediatric patients at a dose of 1 μg/kg. The commercially available enteral form of clonidine, Catapres® tablets, is often compounded into a powder form by pharmacists to achieve dosage adjustments for administration to pediatric patients. However, the stability and quality of compounded clonidine powder have not been verified. The objectives of this study were to formulate a 0.2 mg/g oral clonidine hydrochloride powder and assess the stability and physical properties of this compounded product in storage.

**Methods:**

A 0.2 mg/g clonidine powder was prepared by adding lactose monohydrate to crushed and filtrated clonidine tablets. The powder was stored in polycarbonate amber bottles or coated paper packages laminated with cellophane and polyethylene. The stability of clonidine at 25 °C ± 2 °C and 60% ± 5% relative humidity was examined over a 120-d period in “bottle (closed),” “bottle (in use),” and “laminated paper” storage conditions. Drug dissolution and powder X-ray diffraction analysis were conducted to assess physicochemical stabilities. Validated liquid chromatography-diode array detection was used to detect and quantify clonidine and its degradation product, 2,6-dichloroaniline (2,6-DCA).

**Results:**

Clonidine content was maintained between 90.0 and 110.0% of the initial contents in all packaging and storage conditions. After 120 d of storage, 2,6-DCA was not detected, and no crystallographic and dissolution changes were observed.

**Conclusions:**

Compounded clonidine powder stability was maintained for 120 d at 25 °C ± 2 °C and 60% ± 5% relative humidity. This information may contribute to the management of clonidine compounded powder in community and hospital pharmacies in Japan.

**Supplementary Information:**

The online version contains supplementary material available at 10.1186/s40780-021-00214-x.

## Background

Clonidine hydrochloride is indicated for the treatment of hypertension [1]. In pediatric intensive care units, off-label uses of clonidine include sedation and dexmedetomidine withdrawal [2]. For pediatric administration, 75- or 150-μg clonidine tablets require compounding for dose adjustment. However, clonidine compounding methods, such as crushing tablets and administering the drug orally in powder form, have not been optimized for safe and accurate administration. Therefore, pediatric patients are at risk of receiving poor-quality compounded medication, potentially jeopardizing patient safety and drug efficacy. Furthermore, serious errors involving extemporaneous clonidine preparations have been reported [3–5]. Several adverse events could be linked to the strength and dosage of adult formulations, which are not optimized for pediatric use. To guarantee the efficacy and safety of compounded medicines for pediatric patients, compounded products must be stored and used without changing the chemical stability, crystallization, and dissolution profile.

According to European Medicines Agency (EMA), oral liquid forms are recommended for neonates and children who can swallow and accept enteral feeding [6]. However, most enteral prescriptions for pediatric patients in Japan were prescribed and dispensed as powders or multiparticulates. Recent survey results from 328 pediatric hospitals in Japan revealed that 9.2% of facilities conduct clonidine tablet compounding for pediatric patients [7]. Compounded clonidine tablet stability data are not provided by manufacturers; therefore, the pharmacotherapeutic quality of compounded clonidine products is not guaranteed. Preferably, hospitals should implement physical property testing of active pharmaceutical ingredients (i.e., stability study, dissolution study) according to International Council for Harmonisation of Technical Requirements for Pharmaceuticals for Human Use (ICH) guidelines [8]. Several studies have established clonidine preparation methods for pediatric patients [9–14]. However, to our knowledge, the stability of clonidine hydrochloride powder prepared by crushing commercially available tablets has not been assessed. An implementable standardized method for assuring the quality of compounded clonidine at pediatric hospitals is required.

Our objectives were to formulate an oral powder form of clonidine hydrochloride in extra fine crystal lactose hydrate at a concentration of 0.2 mg/g and assess its stability and physical properties in “bottle (closed),” “bottle (in use),” and “laminated paper” storage conditions. Additionally, we reviewed and compared current compounding standards for pediatric formulations in Japan and other countries.

## Methods

### Reagents and test solution preparation

All chemicals and solvents were analytical grade. Water for chromatography was obtained from a reverse osmosis system (Merk Millipore, Darmstadt, Germany). Clonidine hydrochloride (target substance) and clonidine degradation product 2,6-dichloroaniline (2,6-DCA), also called clonidine impurity-C [14], were used for reference materials (purity > 99.0%; Sigma-Aldrich, Tokyo, Japan). Lactose monohydrate (Extra fine crystal lactose hydrate “Hoei,” Pfizer Co. Ltd., Tokyo, Japan) was used as a diluting agent. Clonidine hydrochloride (20.0 μg/mL) and 2,6-DCA (20.0 μg/mL) standard solutions were prepared by dissolving 2 mg of the respective substances in 100 mL of 50% (v/v) methanol/water. Clonidine hydrochloride test solutions were prepared by dissolving 10 mg of stored compounded powders in 100 mL of 50% (v/v) methanol/water and then diluting with the solvent mixture to obtain final concentrations of 10 μg/mL. Test solutions were prepared in triplicate.

### Clonidine hydrochloride powder compounding

Powder-form clonidine hydrochloride was prepared in the pharmaceutical department in National Center for Child Health and Development, conforming to the Regulations for Buildings and Facilities for Pharmacies. Four hundred 75-μg Catapres® tablets (30 mg of clonidine hydrochloride) were crushed using an automatic pill crusher (KC-HUK2, Konishi Medical Instruments Co., Ltd., Osaka, Japan) at 6000 rpm for 30 s. Crushed tablets were then filtrated using a Japanese Pharmacopoeia certified No. 30 test sieve (Tokyo Screen Co. Ltd., Tokyo, Japan). Extra fine crystal lactose hydrate was added to the sieved powder to make 0.2 mg/g clonidine powder. An automatic mixer (YM-500, Yuyama Co. Ltd., Tokyo, Japan) was used to mix the powder at 620 rotations per min and 20 revolutions per min for 60 s. Compounded clonidine hydrochloride powders were stored at 25 °C ± 2 °C and 60% ± 5% relative humidity (RH) [8].

### Stability study

Clonidine hydrochloride powder stability was assessed in samples withdrawn on days 0, 30, 60, 90, and 120 according to three schedules: (1) the “bottle (closed)” condition, where samples were withdrawn from distinct polycarbonate amber bottles (Yamayu Co. Ltd., Osaka, Japan); (2) the “bottle (in use)” condition [15], where samples were withdrawn from one amber polycarbonate bottle from which 0.1 g was removed daily in a clinical setting; (3) the “laminated paper” condition, where samples were withdrawn from a coated paper package laminated with cellophane and polyethylene (TK70W, Takazono Co. Ltd., Tokyo, Japan). The change in drug content was calculated as (measured concentration/initial concentration) × 100 (%). Changes within 10% of the initial content were considered acceptable changes [16].

### Drug dissolution experiment

Dissolution tests were conducted according to the ICH Japanese Pharmacopoeia 17.6.10 [17] (paddle method; NTR-6400 AC; Toyama Sangyo, Tokyo, Japan) using 900 ml of the dissolution medium maintained at 37 °C ± 0.5 °C and agitated at 50 rpm. We used purified water as the dissolution medium according to the test methods for the original tablet form [1]. At each sampling time, 1.5 mL of the dissolution medium was withdrawn, filtered (0.22-μm syringe filter; Millipore), and stored in test vials at − 20 °C until the analysis. The 0.2 mg/g compounded clonidine powders obtained by crushing tablets were transferred into a dissolution vessel with wax paper. Six samples from each condition, “bottle (closed),” “bottle (in use),” and “laminated paper,” collected after 0, 30, 60, 90, and 120 days of storage were analyzed using the liquid chromatography-diode array detection (LC-DAD) method. The mean dissolution rate of each sample was compared with the sample compounded on day 0.

### Powder X-ray diffraction analysis

Clonidine powders stored in the “bottle (closed)” condition for 60, 90, and 120 days were subjected to powder X-ray diffraction (PXRD) analysis. The PXRD analysis was conducted using a RINT 2000 (Rigaku Co., Tokyo, Japan). The crystallinity of the obtained solid phase was measured at 40 kV voltage, 40 mA current, and a 4°/min scan rate with a nickel filter and a CuKα1 radiation source.

### Clonidine and 2,6-DCA assays

#### Instrumentation and chromatographic conditions

A validated LC method was used to detect clonidine and its degradation product (2,6-DCA), as reported in a previous study [14]. An Ultimate 3000 HPLC system (Thermo Fisher Scientific K.K., Tokyo, Japan) composed of an autosampler, column oven, and diode array detector was used. The autosampler was set at 10 °C, and the column oven was set at 40 °C. Chromatographic separation was performed on a C18 column (Imtakt US-C18 column; 150 × 3.0 mm, 5 μm; Imtakt Co. Ltd., Kyoto, Japan). Eluent A was 10 mM ammonium formate in water, adjusted to pH 4.0 with formic acid, and eluent B was 0.1% formic acid in acetonitrile. An isocratic separation mode was applied at a constant flow rate of 0.4 mL/min from 0 to 10 min, and the composition was maintained at 60% B. The eluents were filtered through a 0.22-μm filter (Merck Millipore, Darmstadt, Germany). The injection volume was set at 5 μL. Detection was performed at 210 nm. Data acquisition, recording, and re-processing were performed using Chromeleon software version 6.80 (Thermo Fischer Scientific K.K., Tokyo, Japan).

#### Linearity, precision, and accuracy

The response linearity was evaluated in triplicate at six concentrations ranging from 2.0 to 20.0 μg/mL for clonidine hydrochloride and 0.01 to 1.0 μg/mL for 2,6-DCA. The calibration curves were validated to ensure that the sample concentrations were within the linear analyte response range. The standard calibration curve fitness was confirmed by back-calculating the calibration standard concentrations. A weighted linear regression (weighting factor: 1/x^2^) method was used to obtain the standard calibration curve and the correlation coefficient. Calibration curve correlation coefficients greater than 0.99 are acceptable for determination using the LC-DAD system. Precision was evaluated in terms of repeatability (intraday) and intermediate precision (interday). Results were expressed as the mean and relative standard deviation (RSD). Repeatability was investigated using six replicate injections of standard solutions, spiked at 10 μg/mL for clonidine and 0.1 μg/mL for 2,6-DCA. The intermediate precision was evaluated by performing six replicate injections on three different days. The accuracy expresses the closeness of agreement between the theoretical and observed values. The accuracy was calculated as (measured concentration/theoretical concentration) × 100 (%). The limit of detection (LOD) and limit of quantification (LOQ) were calculated according to ICH guidelines.

## Results

### LC method and validation

Clonidine and 2,6-DCA retention times were 2.89 min and 6.17 min, respectively (Additional file 1: Fig. S1). The chromatograms showed no interfering peak eluting at the retention times. Weighted linear regression analyses were conducted, and linearity was observed over the examined concentration ranges (2.0–20 μg/mL clonidine; 0.01–1.0 μg/mL 2,6-DCA). The regressions within these ranges had correlation coefficients greater than 0.99, indicating that the method provided a good linear response for clonidine and 2,6-DCA. The regression line slopes and intercepts for clonidine and 2,6-DCA solvent mixtures and clonidine and 2,6-DCA dissolved solutions were not significantly different within the selected concentration ranges. The repeatability (intraday) expressed as %RSD was below 1.0% for clonidine. The recovery was between 98.1 and 109.0%. The method was demonstrated to be sufficiently accurate considering the required specifications for clonidine and 2,6-DCA content (±10.0%). For 2,6-DCA, the LOD was 0.001 μg/mL, and LOQ was 0.01 μg/mL (a signal-to-noise ratio of 13 for an average of six replicates), indicating that the established LC-DAD assay was suitable for the impurity analysis.

### Stability study

The stability study results are presented in Table 1. Upon storage at 25 °C ± 2 °C and 60% ± 5% RH, the clonidine content remained within 90–100% of the initial content over the 120 d storage period in the “bottle (closed),” “bottle (in use),” and “laminated paper” conditions. Representative chromatograms of compounded clonidine products at day 0 and day 120 under each of the three storage conditions are shown in Additional file 2: Fig. S2. No degradation of clonidine (2,6-DCA) was observed during the investigation period.

**Table 1:** Compounded clonidine stability

Study methods	Storage conditions	Storage container	Test periods (days)				
			0	30	60	90	120
Bottle (closed)	25 °C ± 2 °C/60% ± 5%RH	Amber/PC bottle	100.0%	99.2 ± 1.0%	98.8 ± 3.0%	99.1 ± 1.4%	99.3 ± 2.7%
Bottle (in use)		Amber/PC bottle	100.0%	98.9 ± 0.9%	101.0 ± 3.0%	99.7 ± 0.7%	98.6 ± 3.1%
Laminated paper		Amber/CP laminated paper	100.0%	99.9 ± 1.2%	99.4 ± 2.8%	101.9 ± 2.5%	99.2 ± 1.7%

*PC* polycarbonate, *CP* cellophane and polyethylene. Clonidine concentrations are presented as percentages of the day 0 content (100.0%)

### Impurity study

Impurities, including 2,6-DCA, were not observed in the compounded powders on the reference chromatogram at 210 nm (Fig. S2).

### PXRD analysis

**Fig. 1:**
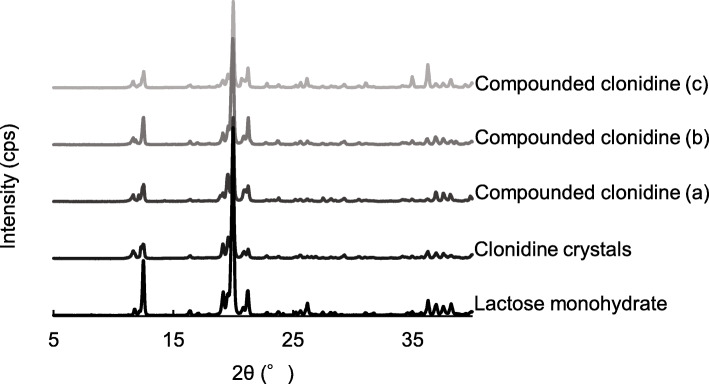
Powder X-ray diffractometry of clonidine stored in the “bottle (closed)” condition for 60 days **(a)**, 90 days **(b)**, and 120 days **(c)**

The clonidine crystals showed characteristic peaks at 2θ = 11.8, 19.2, and 21.3°. The same peaks were observed in compounded clonidine powders stored in the “bottle (closed)” condition for 60, 90, and 120 days, showing no crystallographic changes over the storage period (Fig. 1).

### Dissolution test

**Fig. 2:**
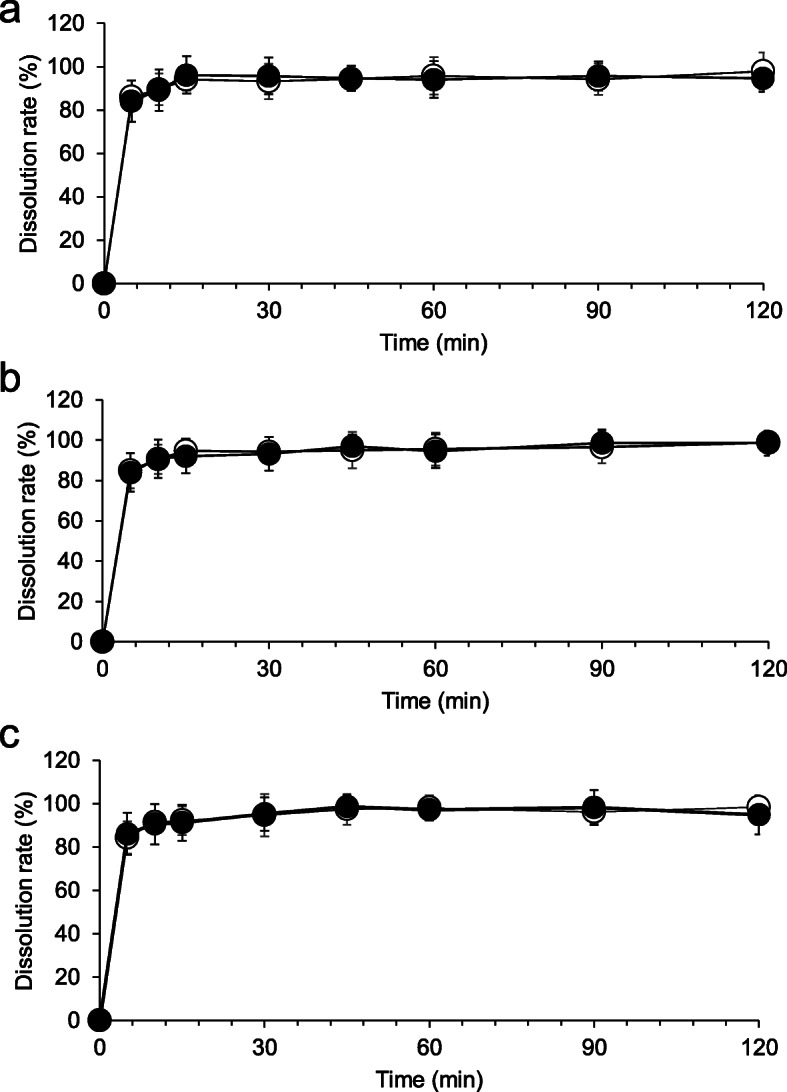
Dissolution profiles of compounded clonidine formula in water stored in the “bottle (closed)” condition **(a)**, “bottle (in use)” condition **(b)**, and “laminated paper” condition **(c)**

All compounded powders tested in water exhibited prompt and complete clonidine dissolution, and the total amount of the drug dissolved in 15 min. No significant variability in dissolution profiles was observed among stored compounded forms and the crushed tablets at day 0 (Fig. 2).

## Discussion

Many commercially available enteral formulations are not suitable for pediatric patients; thus, adult formulations are often compounded by pharmacists and/or caregivers in clinical settings [18], resulting in off-label and unlicensed administration of medicine for pediatric patients. Pediatric formulation development is supported by several stakeholders, such as the World Health Organization [19], EMA [20], US Food and Drug Administration (FDA) [21], Health Canada [22], and European Pediatric Formulation Initiative [23]. Pediatric drug formulation challenges and progress were reviewed by Batchelor et al. [24] in 2015, and an urgent need to improve standardization in compounded enteral formulations to increase safety and preparation compliance was reported. However, most tested pediatric formulations were liquids [25], and the stability and safety of solid oral compounded products have not been reported [26].

A literature review by Hanning et al. [27] also noted that the use of solid oral clonidine formulations for pediatric sedation has not been reported; however, liquid oral clonidine formulations are used for non-parenteral administration in a pediatric intensive care unit setting, offering dose flexibility. The stability of a liquid clonidine formulation was maintained at room temperature for over 270 days in a 50-mg/mL extemporaneous aqueous clonidine hydrochloride solution containing saccharin, raspberry, and methylparaben and buffered at pH 5 [12]. Clonidine also remained stable over 90 days in tablet-dissolved solution stored at 25 °C in amber glass and polyethylene terephthalate bottles [9]. The stability of a 20 mg/mL clonidine hydrochloride suspension prepared by crushing clonidine tablets in simple syrup was maintained for 35 days stored at 4 °C in an amber plastic bottle [13].

Lactose hydrates and corn starch are used as typical diluting agents in routine clinical practice in Japan, but not in other countries. Thus, there is a lack of information on the stability of compounded products diluted by these agents. Here we conducted a stability study using 0.2 mg/g clonidine hydrochloride oral powder that was prepared from commercially available clonidine hydrochloride tablets and extra fine crystal lactose hydrate. Our results showed that the clonidine content did not decrease, and clonidine-related impurities, including the degradation substance (2,6-DCA), were not detected in the LC-DAD assay over the 120-d storage period. Moreover, no differences in clonidine content or structure were observed after storage in open or closed polycarbonate containers or laminated packages. However, because our study did not investigate treatment outcomes or adverse effects experienced by patients, further evaluation of compounded clonidine powder drug efficacy and safety is needed.

In the EU and US, there is a move toward patient-centered formulation development for pediatric enteral medications, which is reflected in the EMA and FDA legislation. Pharmaceutical companies are now required to provide strategies for new, age-appropriate drug development [6, 28]. Furthermore, policies on compounding and quality standards for compounded products and practices, similar to the national compounding protocols established in other countries [29–32], are needed in Japan to compensate for the lack of development of age-appropriate pediatric dosage forms.

Regarding an appropriate approach for the standardization of drug compounding for pediatric patients in Japan, we suggest following protocols similar to those instated by the European Directorate for the Quality of Medicines and HealthCare (EDQM). The EDQM supports the implementation and monitoring of quality standards for medicines and their safe use [33]. In 2013, the EDQM launched the European Paediatric Formulary [34], which provides a publicly available database for extemporaneous pediatric medicine formulations. The formulary is a compilation of monographs for the preparation of such formulations based on the best approaches currently available in national or regional formularies within Europe. The information can be viewed online freely, and four monographs were already available (hydrochlorothiazide 0.5 mg/mL oral solution, sotalol hydrochloride 20 mg/mL oral solution, and furosemide 2 mg/mL oral solution). The EQDM will continue to release monographs on new pediatric formulations elaborated by the PaedForm Working Party [35]. The information included in the monographs includes formulation, quantitative composition, production method, quality control, and storage. For the standardization of drug compounding for pediatric patients in Japan, a similar approach may provide a solution.

In this study, we assessed the stability and physicochemical properties of clonidine powder prepared from the commercially available tablet form. The clonidine content in the compounded powder was maintained for the entirety of the storage period (120 d) in each of the tested storage conditions: “bottle (closed),” “bottle (in use),” and “laminated paper” storage. This established compounding method guarantees the quality of pediatric clonidine formulations and may contribute to the standardization of clonidine compounding in Japanese hospitals.

As a limitation of this study, corn starch and powdered lactose that is used as diluents in other facilities were not examined. Further study for ensuring physio-chemical stability when it formulates in other diluents will be needed.

## Conclusion

Clonidine hydrochloride powder prepared from commercially available tablets remains stable for 120 d at 25 °C ± 2 °C and 60% ± 5% RH in closed bottles, bottles opened daily, and packaged storage conditions.

## Supplementary Information

**Additional file 1: Figure S1** Chromatograms of 10 μg/mL clonidine (a), 10 μg/mL clonidine impurity 2,6-dichloroaniline (b), and solvent mixture (c).

**Additional file 2: Figure S2** Chromatograms of compounded clonidine in the bottle “closed” condition on day 0 (a) and day 120 (b), in the “bottle (in use)” condition on day 0 (c) and day 120 (d), and in the “laminated paper” condition on day 0 (e) and day 120 (f).

## Abbreviations

EMA: European Medicines Agency
ICH: International Council for Harmonisation of Technical Requirements for Pharmaceuticals for Human Use
2,6-DCA: 2,6-dichloroaniline
LC-DAD: liquid chromatography-diode array detection
PXRD: powder X-ray diffraction
RSD: relative standard deviation
LOD: limit of detection
LOQ: limit of quantification
FDA: U.S. Food and Drug Administration
EDQM: European Directorate for the Quality of Medicines and HealthCare
PC: polycarbonate
CP: cellophane and polyethylene

## References

[1] Product Information: Catapres(R) oral tablets, clonidine HCl oral tablets. Boehringer Ingelheim Pharmaceuticals, Inc. (per Manufacturer), Ridgefield, CT, 2012.

[2] Gowing L, Farrell MF, Ali R, White JM (2016). Alpha2-adrenergic agonists for the management of opioid withdrawal. Cochrane Database Syst Rev.

[3] Erickson SJ, Duncan A (1998). Clonidine poisoning--an emerging problem: epidemiology, clinical features, management and preventative strategies. J Paediatr Child Health.

[4] Kappagoda C, Schell DN, Hanson RM, Hutchins P (1998). Clonidine overdose in childhood: implications of increased prescribing. J Paediatr Child Health.

[5] Suchard JR, Graeme KA (2002). Pediatric clonidine poisoning as a result of pharmacy compounding error. Pediatr Emerg Care.

[6] European Medicines Agency. Guideline on pharmaceutical development of medicines for paediatric use. 2013. Available online: https://www.ema.europa.eu/en/documents/scientific-guideline/guideline-pharmaceutical-development-medicines-paediatric-use_en.pdf. Accessed 22 Apr 2021.

[7] Saito J, Akabane M, Nakamura H, Ishikawa Y, Yamatani A (2019). Oral drug compounding in pediatric patients: a Japanese perspective. J Pharmacol Pharm Res.

[8] ICH. Stability testing of active pharmaceutical ingredients and finished pharmaceutical products. 2009. Available online: https://database.ich.org/sites/default/files/Q1F_Stability_Guideline_WHO_2018.pdf. Accessed 22 Apr 2021.

[9] Ensom MH, Décarie D (2014). Stability of extemporaneously compounded clonidine in glass and plastic bottles and plastic syringes. Can J Hosp Pharm.

[10] Ma C, Decarie D, Ensom MH (2014). Stability of clonidine suspension in oral plastic syringes. Am J Health Syst Pharm.

[11] Levinson ML, Johnson CE (1995). Stability of an extemporaneously compounded clonidine hydrochloride oral liquid. Am J Hosp Pharm.

[12] de Goede AL, Boedhram RR, Eckhardt M, Hanff LM, Koch BC, Vermaat CH, Vermes A (2012). Development and validation of a paediatric oral formulation of clonidine hydrochloride. Int J Pharm.

[13] Sauberan JB, Phuong P, Ilog ND, Rossi SS (2016). Stability and osmolality of extemporaneously prepared clonidine Oral liquid for neonates. Ann Pharmacother.

[14] Potier A, Voyat J, Nicolas A (2018). Stability study of a clonidine oral solution in a novel vehicle designed for pediatric patients. Pharm Dev Technol.

[15] European Medicines Agency. Note for guidance on in-use stability testing of human medicinal products. 2001. Available online: https://www.ema.europa.eu/en/documents/scientific-guideline/note-guidance-use-stability-testing-human-medicinal-products_en.pdf. Accessed 22 Apr 2021.

[16] Allen LV Jr. Bassani GS, Elder EJ, Parr AF. Strength and Stability Testing for Compounded Preparations. U.S. Pharmacop. 2014. Available online: https://www.usp.org/sites/default/files/usp/document/FAQs/strength-stability-testing-compounded-preparations.pdf. Accessed 22 Apr 2021.

[17] The ministry of health, labour and welfare, The Japanese pharmacopoeia seventeenth edition. 2016. Available online: https://www.pmda.go.jp/files/000217650.pdf Accessed 22 Apr 2021.

[18] Saito J, Akabane M, Ishikawa Y, Iwahashi K, Nakamura H, Yamatani A (2020). Retrospective survey of compounded medications for children in Japan. Eur J Pharm Biopharm.

[19] Gerrard SE, Walsh J, Bowers N, Salunke S, Hershenson S (2019). Innovations in pediatric drug formulations and administration technologies for low resource settings. Pharmaceutics..

[20] van Riet-Nales DA, Kozarewicz P, Aylward B, de Vries R, Egberts TC, Rademaker CM, Schobben AF (2017). Paediatric drug development and formulation design-a European perspective. AAPS PharmSciTech.

[21] Gadge PM, Kenjale PP, Pokharkar VB, Gaikwad VL (2019). Global pediatric regulations: an overview. Ther Innov Regul Sci.

[22] Hepburn CM, Gilpin A, Autmizguine J, Denburg A, Dupuis LL, Finkelstein Y, Gruenwoldt E, Ito S, Jong G', Lacaze-Masmonteil T, Levy D, Macleod S, Miller SP, Offringa M, Pinsk M, Power B, Rieder M, Litalien C. Improving paediatric medications: a prescription for Canadian children and youth. Paediatr Child Health 2019;24:333–339, 5, DOI: 10.1093/pch/pxz079. 10.1093/pch/pxz079 PMC6656949 31379437

[23] Ivanovska V, Rademaker CM, van Dijk L, Mantel-Teeuwisse AK (2014). Pediatric drug formulations: a review of challenges and progress. Pediatrics..

[24] Batchelor H, Salunke S, Tuleu C (2015). Formulating better medicines for children-reflections. Int J Pharm.

[25] Conroy S (2003). Extemporaneous (magistral) preparation of oral medicines for children in European hospitals. Acta Paediatr.

[26] Rood JM, Engels MJ, Ciarkowski SL, Wagenknecht LD, Dickinson CJ, Stevenson JG (2014). Variability in compounding of oral liquids for pediatric patients: a patient safety concern. J Am Pharm Assoc.

[27] Hanning SM, Orlu Gul M, Toni I, Neubert A, Tuleu C (2017). A mini-review of non-parenteral clonidine preparations for paediatric sedation. J Pharm Pharmacol.

[28] U.S. Food and Drug Administration. Pediatric Study Plans: Content of and Process for Submitting Initial Pediatric Study Plans and Amended Initial Pediatric Study Plans. 2020. Available online: https://www.fda.gov/regulatory-information/search-fda-guidance-documents/pediatric-study-plans-content-and-process-submitting-initial-pediatric-study-plans-and-amended. Accessed 22 Apr 2021.

[29] Jackson M, Lowey A (2010). Handbook of extemporaneous formulation: a guide to pharmaceutical compounding.

[30] The Hospital for Sick Children (SickKids), Online Database. 2014. Available online: http://www.sickkids.ca/Pharmacy/Compounding-Service/index.html. Accessed 22 Apr 2021.

[31] Canadian Society of Hospital Pharmacist, Compounding: Guidelines for Pharmacies. 2014. Available online: https://cshp.ca/compounding-guidelines-pharmacies Accessed 22 Apr 2021.

[32] Pharmacy Board of Australia, Guidelines on Compounding of Medicines. 2015. Available online: https://www.pharmacyboard.gov.au/documents/default.aspx?record=WD15%2F16205&dbid=AP&chksum=3QlnioMt0DhI0PsjaoB83A%3D%3D. Accessed 22 Apr 2021.

[33] European Medicines Agency. European Directorate for the Quality of Medicines and HealthCare (EDQM) of the Council of Europe. Available online: https://www.ema.europa.eu/en/partners-networks/international-activities/multilateral-coalitions-initiatives/european-directorate-quality-medicines-healthcare-edqm-council-europe. Accessed 22 Apr 2021.

[34] Council of Europe, European Paediatric Formulary. Available online: https://paedform.edqm.eu/home Accessed 22 Apr 2021.

[35] Dirk L. A pan-European Paediatric Formulary. 2016. https://www.edqm.eu/sites/default/files/poster_pheur_pan_european_paediatric_formulary_2016.pdf. Accessed 22 Apr 2021.

