# Supplementary figures and images for: Stability of clonidine hydrochloride in an oral powder form compounded for pediatric patients in Japan
Source: J Pharm Health Care Sci. 2021 Sep 1;7:31. doi: 10.1186/s40780-021-00214-x (PMC8408926; doi:10.1186/s40780-021-00214-x)

**a**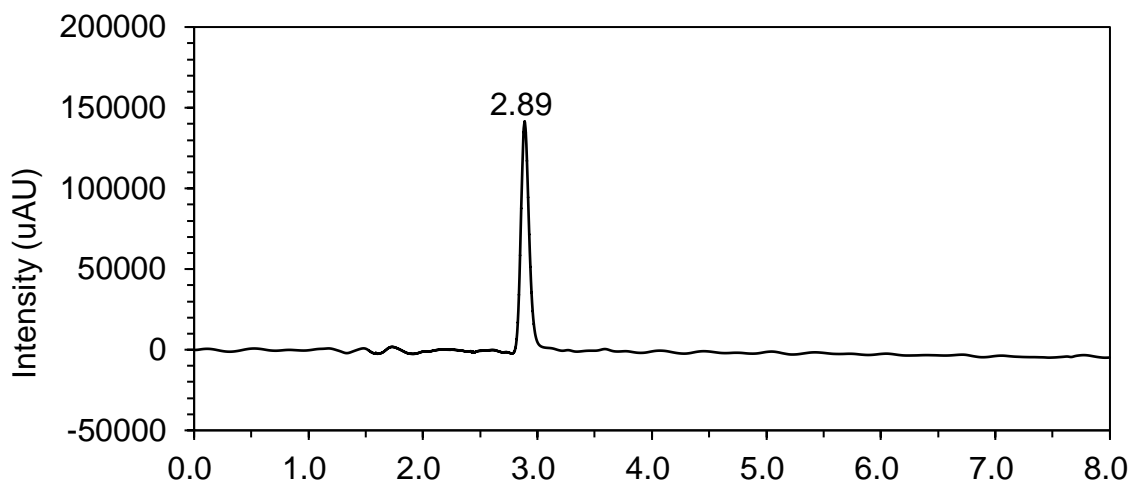**b**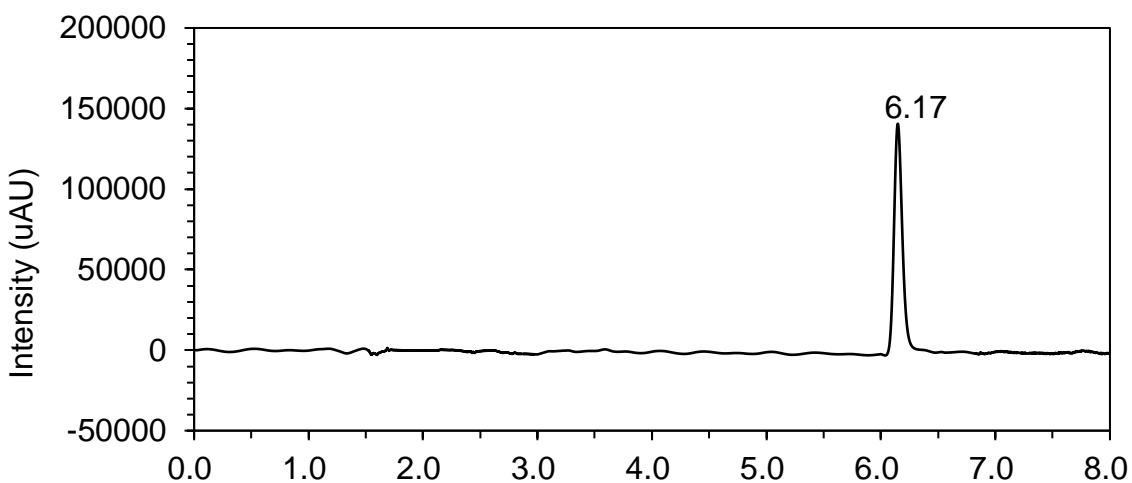**c**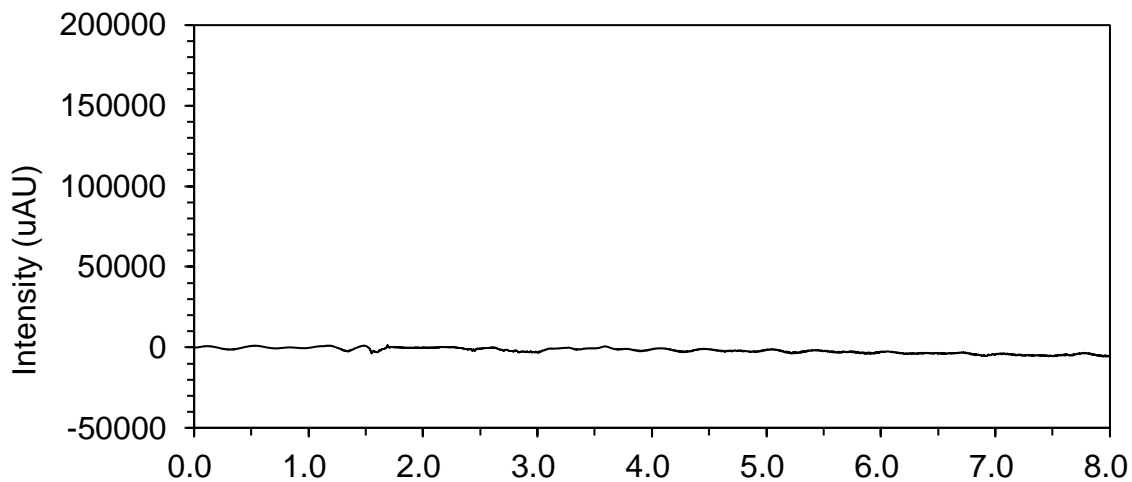

Supplement: Supplementary file 1 — Additional file 1: Figure S1 Chromatograms of 10 μg/mL clonidine (a), 10 μg/mL clonidine impurity 2,6-dichloroaniline (b), and solvent mixture (c). [file 40780_2021_214_MOESM1_ESM.pdf]

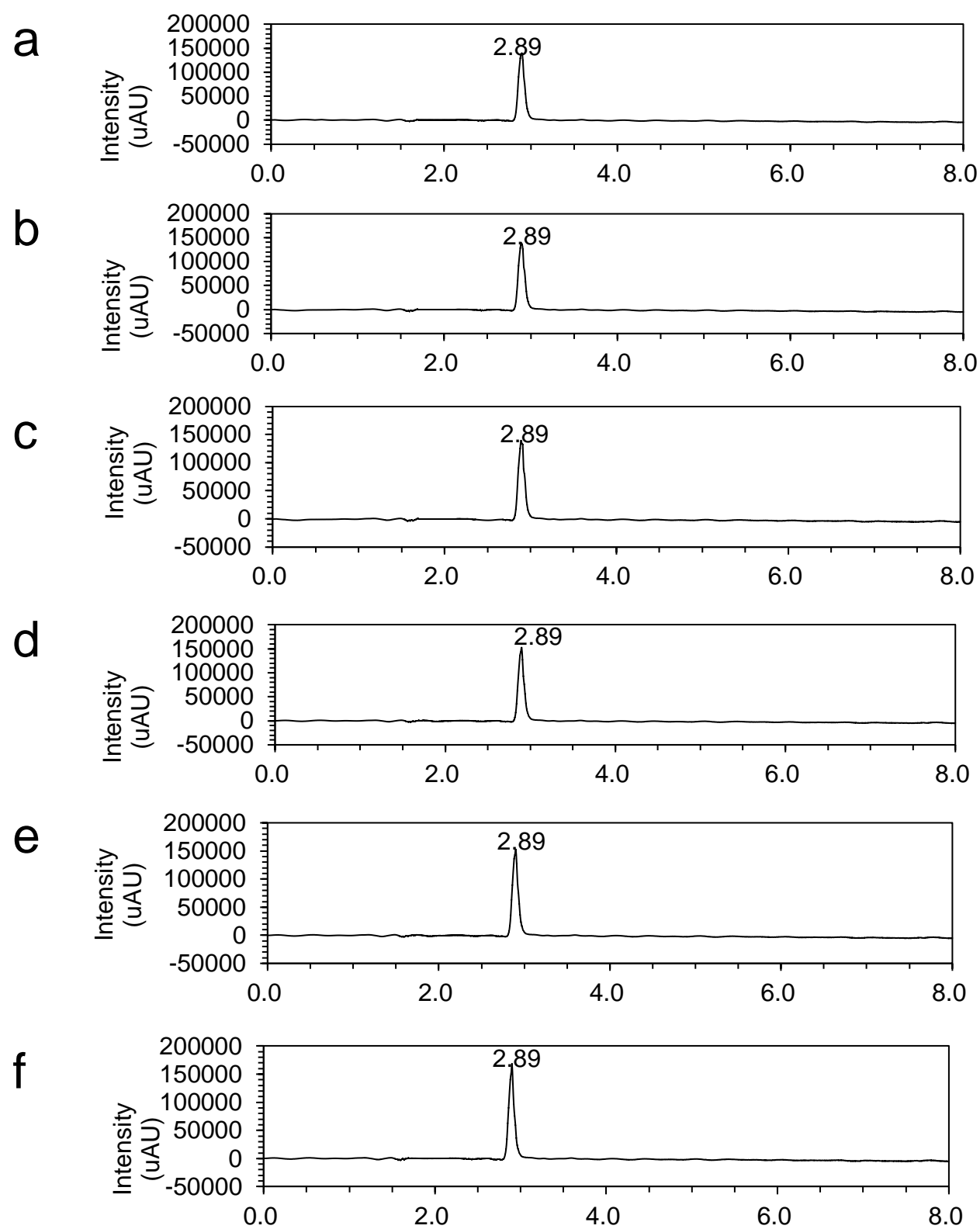

Supplement: Supplementary file 2 — Additional file 2: Figure S2 Chromatograms of compounded clonidine in the bottle “closed” condition on day 0 (a) and day 120 (b), in the “bottle (in use)” condition on day 0 (c) and day 120 (d), and in the “laminated paper” condition on day 0 (e) and day 120 (f). [file 40780_2021_214_MOESM2_ESM.pdf]
